# Rare Case of a Well-Differentiated Paratesticular Sarcoma of the Spermatic Cord in a 60-Year-Old Patient

**DOI:** 10.1155/2017/7903242

**Published:** 2017-03-02

**Authors:** Marwen Benna, Semia Zarraâ, Asma Belaïd, Aziz Cherif, Lotfi Kochbati, Mohammed Chebil, Farouk Benna

**Affiliations:** ^1^Department of Radiation Therapy, Salah Azaiez Institute, Tunis, Tunisia; ^2^Department of Urology, Charles Nicole Hospital, Tunis, Tunisia

## Abstract

*Introduction*. Liposarcomas are tumors that occur mostly in the retroperitoneum. Of all liposarcomas only 3 to 7% are found in the paratesticular region. The spermatic cord is the main site of origin in these cases. The patients ages range from 50 to 60 years. This malignant disease can result in a loss of fertility aside from life-threatening sequelae.* Case*. We present a case of a liposarcoma of the paratesticular region. A 60-year-old man was referred with a painless mass in the scrotum and the right inguinal region. The patient underwent surgery and the mass was removed along with the right testis, the spermatic cord, and the soft tissues to the internal inguinal ring. Histopathological examination found a well-differentiated liposarcoma of 80⁎80 mm. The surgical margins were negative. The adjuvant treatment consisted in radiation therapy of the right inguinoscrotal area to the dose of 54 Gray, 2 Gy per session, 5 times a week.* Conclusion*. Paratesticular liposarcomas are rare tumors. Surgery with large margin resections was the main treatment in all reported cases. The adjuvant treatment is still unclear especially when the surgical margins are negative. The main factor that indicated this adjuvant treatment was the size of the tumor and the histologic subtype.

## 1. Introduction

Primary paratesticular tumors are rare, only accounting for 7% to 10% of all intrascrotal tumors. The mean age in these cases is 50 to 60 ranging from 16 to 82 years [1]. All of these cases have been reported separately and there is no evidence of a hereditary disorder causing them. There is no known environmental cause to these cases due to their rarity. These tumors are usually symptomatic, large, and rapid growth tumors [2]. There is an exceptional reported case of a 30 cm tumor in a patient who refused treatment for years due to intellectual disability making surgical treatment most complex and indicating adjuvant treatment [3].

In adults, more than 75% of paratesticular tumors arise from the spermatic cord, with 20% being liposarcomas. Tumor grade, stage, histological type, and lymph node involvement are independently predictive of prognosis [2].

Low-grade liposarcomas have a good prognosis, whereas high grade tumors often develop metastases and have a significant tumor-related mortality [2, 6].

## 2. Case

A 60-year-old male patient noticed a scrotal swelling during the past 6 months. He had no urinary tract symptoms. The mass was indolent and slowly growing. Physical examination revealed a firm mass, distinct from the testicle which was of a normal size and consistency. The internal inguinal ring could not be reached and the mass was nonreducible.

**Figure 1:**
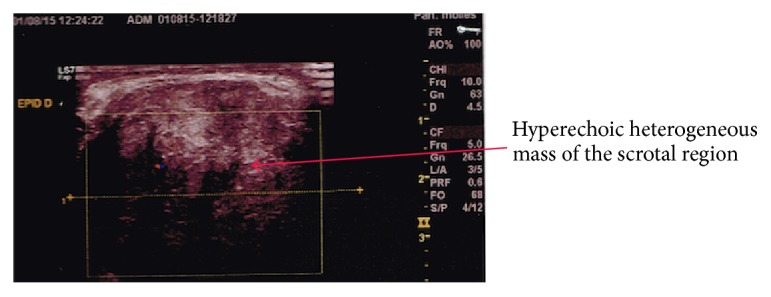
Ultrasonography showing a mass originating from the epidemic right chord.

Scrotal ultrasonography revealed a large right scrotal hyperechogenic and heterogeneous measuring 80 mm as seen in Figure 1. Both testicles were strictly normal. Testicular tumor markers (AFP, B-HCG) were strictly normal. Thoracoabdominal scan showed no regional or distant metastasis.

The patient underwent surgery. Through inguinal incision, the mass was removed in bloc with the right testis, the spermatic cord, and the soft tissues to the internal inguinal ring. Histopathological examination found a sclerosing variant of a grade 1 well-differentiated liposarcoma of 80*∗*80 mm. The surgical margins were negative.

Due to the histologic subtype and the size of the tumor, adjuvant treatment was indicated. The patient accepted the possible adverse effects of the treatment comprising infertility. The CTV (clinical target volume) was defined by the right inguinal, the right scrotal region, and the surgical scar. The PTV (planning target volume) was defined by a 10 mm margin around the CTV. The prescribed dose was 54 Gy, 2 Gy per session, 5 times a week. The patient had a GII acute radiation dermatitis that was well treated with topic medication.

## 3. Discussion

Due to the rare occurrence of paratesticular liposarcoma, there is no consensus on treatment. Fewer than 200 paratesticular liposarcomas were reported [4]. Like in all known sarcomas, complete resection is the best treatment to date. Radical orchiectomy with high ligation of the spermatic cord at the inguinal ring is advised [4] since it can achieve a resection with sufficient margins. Large excision does not suffice and is associated with early local recurrence [7].

There is no indication for routine lymph node dissection as the locoregional lymph nodes are rarely involved according to published data [5].

Nevertheless, some authors recommend ipsilateral pelvic lymph node dissection as part of the initial treatment due to the risk of late regional reoccurrence [8].

To avoid locoregional failure, most authors considered prophylactic retroperitoneal lymph node dissection or radiation therapy as viable options.

To assess the benefit of extensive lymph node dissection, Banowsky and Shultz reported 101 cases of liposarcomas. 29 prophylactic retroperitoneal lymph node dissections were performed out of which 17 were positive. But this treatment had no demonstrated impact on survival [9].

Due to the morbidity of this surgery and no clear benefit, retroperitoneal lymph node dissection should not be performed unless positive lymph nodes are encountered on CT scans or palpated during surgery [2].

Liposarcomas are the most radiosensitive sarcoma and in some cases remission has been achieved with radiotherapy alone [10]. There are no prospective randomized studies or even large retrospective series advocating the role of adjuvant radiation therapy. However, because of the high propensity of local recurrence following surgery alone, there is increasing consensus that all paratesticular liposarcomas should receive adjuvant radiotherapy [11]. Many authors recommend systematic radiation therapy on the inguinal region [2, 12, 13]. With the most recent techniques, pelvic radiotherapy presents an acceptable morbidity [14] and ipsilateral should be considered regarding prognosis factors which are histological subtype, grade, and size of the tumor >5 cm [15].

## 4. Conclusion

Paratesticular liposarcomas are rare tumors with few available data. The recommended treatment course is radical orchiectomy with high ligation of the spermatic cord at the inguinal ring. Ipsilateral pelvic lymph node dissection is advisable. Further lymph node dissection should be guided by preoperative CT scans and per operative findings. Adjuvant treatment should include radiation therapy guided by extension work-up and histological examination. Areas of interest should include the ipsilateral inguinal region and the ipsilateral pelvic region if no lymph node dissection was performed. Follow-up should be thorough and long term due to the possibility of late recurrence.
